# Dose-Dependent Biphasic Action of Quetiapine on AMPK Signalling via 5-HT7 Receptor: Exploring Pathophysiology of Clinical and Adverse Effects of Quetiapine

**DOI:** 10.3390/ijms23169103

**Published:** 2022-08-14

**Authors:** Motohiro Okada, Kouji Fukuyama, Eishi Motomura

**Affiliations:** Department of Neuropsychiatry, Division of Neuroscience, Graduate School of Medicine, Mie University, Tsu 514-8507, Japan

**Keywords:** Akt, AMPK, astrocyte, connexin43, D-serine, Erk, quetiapine

## Abstract

Recent pharmacological studies indicated that the modulation of tripartite-synaptic transmission plays important roles in the pathophysiology of schizophrenia, mood disorders and adverse reactions. Therefore, to explore the mechanisms underlying the clinical and adverse reactions to atypical antipsychotics, the present study determined the effects of the sub-chronic administration of quetiapine (QTP: 3~30 μM) on the protein expression of 5-HT7 receptor (5-HT7R), connexin43 (Cx43), cAMP level and intracellular signalling, Akt, Erk and adenosine monophosphate-activated protein kinase (AMPK) in cultured astrocytes and the rat hypothalamus, using ultra-high-pressure liquid chromatography with mass spectrometry and capillary immunoblotting systems. QTP biphasically increased physiological ripple-burst evoked astroglial D-serine release in a concentration-dependent manner, peaking at 10 μM. QTP enhanced the astroglial signalling of Erk concentration-dependently, whereas both Akt and AMPK signalling’s were biphasically enhanced by QTP, peaking at 10 μM and 3 μM, respectively. QTP downregulated astroglial 5-HT7R in the plasma membrane concentration-dependently. Protein expression of Cx43 in astroglial cytosol and intracellular cAMP levels were decreased and increased by QTP also biphasically, peaking at 3 μM. The dose-dependent effects of QTP on the protein expression of 5-HT7R and Cx43, AMPK signalling and intracellular cAMP levels in the hypothalamus were similar to those in astrocytes. These results suggest several complicated pharmacological features of QTP. A therapeutically relevant concentration/dose of QTP activates Akt, Erk and AMPK signalling, whereas a higher concentration/dose of QTP suppresses AMPK signalling via its low-affinity 5-HT7R inverse agonistic action. Therefore, 5-HT7R inverse agonistic action probably plays important roles in the prevention of a part of adverse reactions of QTP, such as weight gain and metabolic complications.

## 1. Introduction

Quetiapine (QTP) is one of the most commonly utilised mood-stabilising atypical antipsychotics; it is approved for the treatment of schizophrenia and several types of mood disorders, such as major depression, bipolar depression and mania, in a number of countries [1,2]. Furthermore, off-label uses for a variety of symptoms, including insomnia, agitation, anxiety, dementia, obsessive-compulsive disorder and psychosis in patients with Parkinson’s disease, are also well known [3,4]. Indeed, QTP has been ranked as one of the 100 most commonly prescribed medications in the United States in the past decade due to its wide clinical spectrum [5]. Several meta-analyses and systematic reviews confirmed the findings regarding the clinical effectiveness of QTP [6,7,8,9,10,11,12,13]. Additionally, these reports also suggested the existence of a dose-dependent clinical efficacy spectrum of QTP: unipolar depression—150~300 mg/day, bipolar depression—300~600 mg/day and mania and schizophrenia—more than 600 mg/day [3,6,7,8,9,10,11,12,13]. In contrast to the effectiveness of a high dose of QTP, a daily dose of QTP of 300 mg/day was superior to 600 mg/day with respect to quality of life and metabolic complications [9]. Notably, a dose–response meta-analysis revealed that the dose–response curve of weight gain induced by QTP was approximately bell-shaped and its peak was 1.48 kg at around 600 mg/day [14].

The pathophysiology of the clinical effects and adverse reactions of QTP is not a simple mechanism, since QTP binds various transmitter receptors [15,16] and affects various transmission systems [17,18]. QTP is a potent histamine H1 receptor (H1R) antagonist (H1R: Ki = 11 nM) resembling clozapine (Ki = 1.13 nM), when compared to other atypical antipsychotics [15] (Table 1). QTP presents relatively higher potencies to α1A adrenoceptors and 5-HT2A receptor (5-HT2AR) as antagonists, but lower potencies to dopamine D2 receptor (D2R) antagonism with 5-HT1A receptor (5-HT1AR) partial agonism [15]. According to these unique receptor binding profiles of QTP, it can be speculated that QTP at a dose lower than 50 mg/day acutely induces hypnotic and daytime sedation due to primarily H1R and adrenoceptor antagonism [19]. With an increasing concentration, QTP increases monoamine release via pre-synaptic disinhibition induced by 5-HT2AR suppression in the frontal cortex [17]. Further increasing the QTP concentration, the somatodendritic dopamine D2 receptor (D2R) on dopaminergic neurons in the midbrain is inhibited, resulting in the activation of dopaminergic transmission [17]; however, it has been questioned whether the 5-HT1AR partial agonistic action of QTP contributes as a potential mechanism underlying the pathophysiology of QTP [20].

In spite of these efforts, the features of the clinical action of QTP cannot be fully explained by its receptor binding profiles alone [18]. Recent pharmacodynamic findings have been accumulated to support the possibility that functional abnormalities of tripartite synaptic transmission play important roles in the pathophysiology of mood disorders [26,27,28]. Furthermore, the expression of several monoamine receptors, including 5HT1AR and 5-HT7 receptors (5-HT7R), in astrocytes has been identified [18,29,30,31]. According to the monoaminergic tripartite synaptic transmission hypothesis of mood disorders, it has been suggested that the pharmacodynamic profile of astroglial transmission associated with the hemichannel has a correlation with efficacy in mood disorders [26,28,32]. In particular, several pharmacogenetic studies reported that 5-HT7R inhibition played key roles in the pathophysiology of mood disorders other than schizophrenia [26,33,34,35]. Indeed, a 5-HT7R and 5-HT transporter-inhibiting antidepressant, vortioxetine [36,37], suppresses astroglial L-glutamate release via inhibition of connexin43 (Cx43) trafficking to the plasma membrane [31]. Similar to vortioxetine, mood-stabilising antipsychotics, such as brexpiprazole and lurasidone, which are effective for bipolar depression but not for mania, also suppress astroglial L-glutamate release due to the same mechanism [18,28,38,39,40,41]. It has been demonstrated that 5-HT7R inhibition probably plays an important role in the underlying effects of vortioxetine, brexpiprazole and lurasidone on the suppression of Cx43 trafficking [31,38,39]. Contrarily, several mood-stabilising atypical antipsychotics, such as clozapine, QTP and zotepine, which are effective for a wide spectrum of mood disorders, including unipolar depression, bipolar depression and mania, increase astroglial L-glutamate release via activation of Cx43 trafficking [18,26,28,40,41].

Both antidepressive mood-stabilising antipsychotics, lurasidone and brexpiprazole, are evaluated to be the safest option in patients with a risk of developing metabolic complications, since they are listed among the best atypical antipsychotics associated with metabolic outcomes [14,42]. The mechanisms of similar clinical features between lurasidone and brexpiprazole, which are of low risk for the development of metabolic complication, are also probably involved in the 5-HT7R inverse agonistic action of these atypical antipsychotics [38,39]. In particular, the 5-HT7R inverse agonistic actions of lurasidone and brexpiprazole are speculated to contribute to the suppression of adenosine monophosphate-activated protein kinase (AMPK) signalling, resulting in a low risk of developing metabolic complications [38,39,43]; however, clozapine is also known to be a 5-HT7R inverse agonist [44]. Clozapine acutely inhibits 5-HT7R functions and chronically downregulates 5-HT7R [44], whereas the binding affinity of clozapine to 5-HT7R (Ki = 18 nM) is relatively lower compared to H1R (Ki = 1.1 nM) [22,23] (Table 1). The binding affinity of QTP to 5-HT7R (Ki = 307 nM) is also relatively weak compared to H1R (Ki = 11 nM) [15] (Table 1). The inhibitory action of QTP on 5-HT7R and H1R has been speculated to provide the mechanisms inherent in the antidepressant effects of QTP [45]; however, considering the clinical findings that the antidepressant effect of QTP is predominant at low doses, the low binding affinity of QTP to 5-HT7R possibly contributes to the dose-dependent biphasic effect of high doses of QTP on weight gain, rather than its antidepressive effects. Therefore, the effects of QTP on 5-HT7R remain to be clarified. Based on these aspects, the present study performed several experiments, to clarify the pathophysiology of the upper limit of the therapeutically relevant concentration of QTP associated with 5-HT7R, considering (1) the concentration-dependent effects of QTP on the release of gliotransmitter D-serine through activated astroglial Cx43-containing hemichannel; (2) the concentration-dependent effects of intracellular signalling, including protein kinase B (Akt), extracellular signal-regulated kinase (Erk) and AMPK; (3) finally, the subchronic effects of the systemic administration of QTP on AMPK signalling in the hypothalamus.

## 2. Results

### 2.1. Effects of Intracellular Signalling and QTP on Basal and Artificial Ripple-Burst Evoked Astroglial D-Serine Release

Astrocytes release gliotransmitters via several systems, such as exocytosis [46,47], transporters [48] and hemichannels [49]. During the resting state, the astroglial hemichannel cannot release gliotransmitters due to its low opening probability, whereas depolarisation of the plasma membrane activates hemichannel activity, resulting in the release of gliotransmitter through activated astroglial hemichannels [27,50,51,52]. To clarify the concentration-dependent effects of QTP on physiological astroglial D-serine release, the present study determined the basal and ripple-burst (which is observed during sleep spindle burst and contributes to cognitive function) evoked astroglial D-serine release (detailed method of artificial ripple-burst evoked stimulation is described in Section 4.3).

#### 2.1.1. Effects of Intracellular Signalling on Basal and Artificial Ripple-Burst Evoked Astroglial D-Serine Release

**Figure 1:**
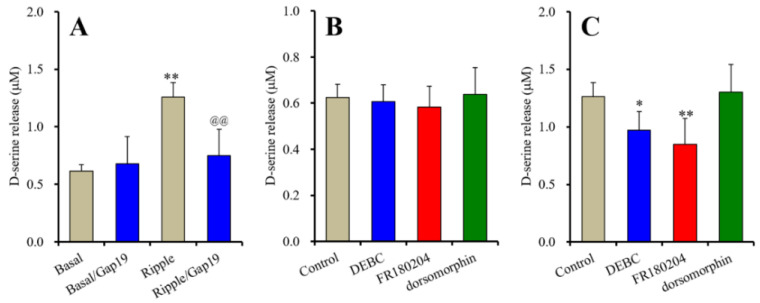
Effects of 10 μM TAT-conjugated Gap19 (Gap19), connexin43 (Cx43) inhibitor, on acute artificial ripple-burst evoked astroglial D-serine release (**A**), effects of intracellular signalling inhibitors on basal (**B**) and ripple-evoked astroglial D-serine releases (**C**). In Panel A, during ripple-evoked stimulation (100 sets), the cultured astrocytes were incubated in artificial cerebrospinal fluid (ACSF) with or without (control) 10 μM Gap19. In Panel B: the cultured astrocytes were subchronically (for 7 days) administrated by intracellular signalling inhibitors, protein kinase B (Akt) inhibitor, 10-[4-(N,N-diethylamino)butyl]-2-chlorophenoxazine hydrochloride (DEBC: 10 μM), extracellular signal-regulated kinase (Erk) inhibitor, 5-(2-Phenyl-pyrazolo[1,5-a]pyridin-3-yl)-1H-pyrazolo[3,4-c]pyridazin-3-ylamine (FR180204: 20 μM) and adenosine monophosphate-activated protein kinase (AMPK) inhibitor (dorsomorphin: 10 μM). In Panel C: after subchronic applications of 10 μM DEBC, 20 μM FR180204 or 10 μM dorsomorphin, the cultured astrocytes were stimulated by ripple-burst evoked stimulation (100 sets). Ordinates indicate the mean ± standard deviation (SD) of extracellular D-serine level (μM) (*n* = 6). * *p* < 0.05, ** *p* < 0.01; relative to control, @@ *p* < 0.01; relative to ripple-burst evoked release (Gap19 free) by one-way analysis of variance (ANOVA) with Tukey’s post-hoc test.

In our previous study, acute artificial ripple-burst evoked stimulations (100 sets) did not affect astroglial L-glutamate release [51,53], whereas astroglial D-serine release was increased by acute artificial ripple-burst evoked stimulations (100 set) (Figure 1A). Basal astroglial D-serine release was not affected by 10 μM TAT-conjugated Gap19 (Gap19), a selective Cx43-containing hemichannel inhibitor, whereas ripple-burst evoked D-serine release was inhibited by 10 μM Gap19 (Figure 1A).

An Akt inhibitor, 10-[4-(N,N-diethylamino)butyl]-2-chlorophenoxazine hydrochloride (DEBC: 10 μM), Erk inhibitor, 5-(2-Phenyl-pyrazolo[1,5-a]pyridin-3-yl)-1H-pyrazolo[3,4-c]pyridazin-3-ylamine (FR180204: 20 μM) [18,31,39,40,52] and AMPK inhibitor, dorsomorphin (10 μM) [54], also did not affect basal astroglial D-serine release (Figure 1B), whereas ripple-burst evoked D-serine release was inhibited by 10 μM DEBC and 20 μM FR180204, but was not affected by 10 μM dorsomorphin (Figure 1C).

These results suggest that during the resting state, astroglial D-serine is not released through the astroglial Cx43-containing hemichannel; however, during physiological repetitive firing, such as ripple bursts, astrocytes release D-serine through the activated astroglial hemichannel. Furthermore, Akt and Erk signalling play important roles in the electrophysiological activation process of the astroglial hemichannel rather than AMPK.

#### 2.1.2. Concentration-Dependent Effects of QTP on Astroglial D-Serine Release

**Figure 2:**
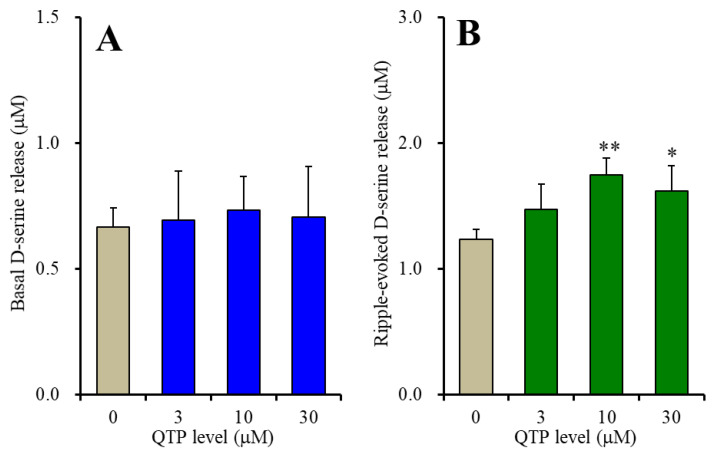
Concentration-dependent effects of subchronic administration of quetiapine (QTP) on basal (**A**) and artificial ripple-burst evoked (**B**) astroglial D-serine releases. Ordinate indicates the mean ± SD of extracellular D-serine level (μM) (*n* = 6). Abscissa indicates QTP concentration (μM). * *p* < 0.05, ** *p* < 0.01; relative to control (QTP free) by one-way ANOVA with Tukey’s post-hoc test.

The therapeutically relevant serum concentration of QTP was clinically reported to approximately range from 0.3 μM to 3 μM [55,56]. A recent study using primary cultured astrocytes demonstrated that basal astroglial L-glutamate release increased by higher than 10 μM QTP, whereas astroglial L-glutamate release through the activated hemichannel was increased by higher than 1 μM QTP [18]. Based on these clinical and preclinical findings, in the present study, cultured astrocytes were administrated with the upper limit concentration (3 μM) and supratherapeutic concentration (10 and 30 μM) of QTP for 7 days [18]. Subchronic administration of QTP for 7 days did not affect basal astroglial D-serine release (Figure 2A). After subchronic exposure to QTP, ripple-burst evoked stimulation (100 sets) was enhanced by QTP, in a biphasically concentration-dependent manner [F (3,20) = 12.9 (*p* < 0.01)], since the concentration response to QTP of ripple-burst evoked D-serine release was approximately bell-shaped and its peak concentration was 10 μM (D-serine induced by 10 μM QTP was higher than that by 3 μM and 30 μM QTP) (Figure 2B).

**Figure 3:**
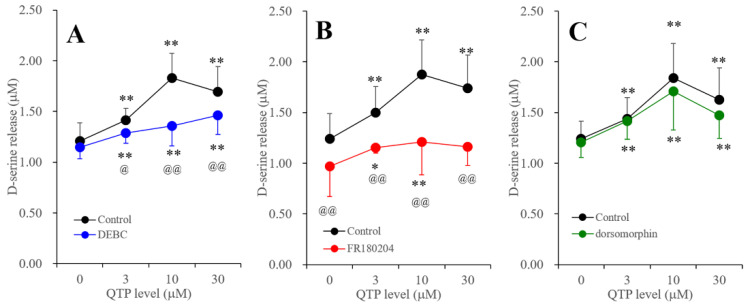
Interaction between subchronic administration of QTP and intracellular signalling inhibitors, 10 μM DEBC (**A**), 20 μM FR180204 (**B**) and 10 μM dorsomorphin (**C**) on astroglial ripple-burst evoked D-serine release. Ordinates indicate the mean ± SD of extracellular D-serine level (μM) (*n* = 6). Abscissa indicates QTP concentration (μM). * *p* < 0.05, ** *p* < 0.01; relative to QTP free, @ *p* < 0.05, @@ *p* < 0.01; relative to control (inhibitor free) by two-way ANOVA with Tukey’s post-hoc test.

To clarify the mechanisms of the biphasically concentration-dependent effects of QTP on ripple-burst evoked D-serine from astrocytes, after the subchronic administration of QTP with 10 μM DEBC, 20 μM FR180204 or 10 μM dorsomorphin, the ripple-burst evoked astroglial D-serine release was determined. The stimulatory effects of QTP on astroglial ripple-burst evoked D-serine release were not affected by the AMPK inhibitor (Figure 3C). Both DEBC [F_QTP_ (3,30) = 112.0 (*p* < 0.01), F_DEBC_ (1,10) = 4.9 (*p* > 0.05), F_QTP*DEBC_ (3,30) = 23.0 (*p* < 0.01)] and FR180204 [F_QTP_ (3,30) = 39.4 (*p* < 0.01), F_FR180204_ (1,10) = 10.1 (*p* < 0.01), F_QTP*FR180204_ (3,30) = 9.5 (*p* < 0.01)] suppressed the ripple-burst evoked D-serine release (Figure 3A,B).

The Erk inhibition by FR180204 appeared to show greater suppression than that of DEBC (Akt inhibitor). During the inhibition of Erk by FR180204, the biphasic effects of QTP remained to be observed, whereas DEBC abolished the bell-shaped ripple-burst evoked D-serine release induced by QTP. These results suggest that the concentration-dependent biphasic action of QTP on ripple-burst evoked astroglial D-serine release is possibly mediated by Akt signalling.

### 2.2. Concentration-Dependent Effects of QTP on Protein Expression in Astrocyte

#### 2.2.1. Effects of QTP on Intracellular Signalling in Astrocytes

**Figure 4:**
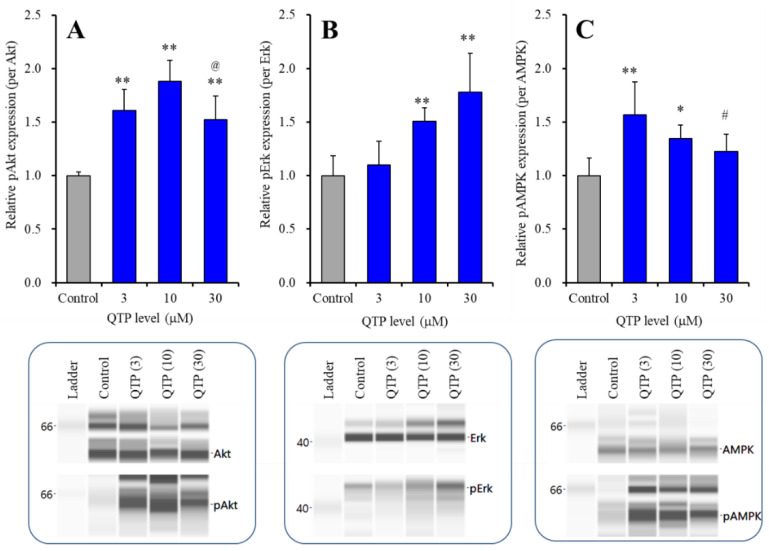
Concentration-dependent effects of subchronic administration (7 days) of QTP on protein expression of pAkt (**A**), pErk (**B**) in the plasma membrane fraction and pAMPK (**C**) in the cytosol fraction of cultured astrocytes. In upper side histograms, ordinate: mean ± SD (*n* = 6) of the relative protein level of pAkt, pErk and pAMPK per Akt, Erk and AMPK, respectively. Abscissa: QTP level (μM). * *p* < 0.05, ** *p* < 0.01: relative to control (QTP free), @ *p* < 0.05: relative to 10 μM QTP in panel A; # *p* < 0.05: relative to 3 μM QTP in panel C by one-way ANOVA with Tukey’s post-hoc test. Lower side panels indicate their pseudo-gel images, using capillary immunoblotting.

Subchronic administration of QTP for 7 days did not affect the protein expression of Akt, Erk or AMPK (Figure 4). Contrarily, subchronic administration of QTP increased the phosphorylation of Akt, Erk and AMPK (Figure 4). Both pAkt [F (3,20) = 25.6 (*p* < 0.01)] and pAMPK [F (3,20) = 8.1 (*p* < 0.01)] were increased by subchronic administration in a biphasically concentration-dependent manner, and the peak QTP levels for pAkt and pAMPK were 3 μM and 10 μM, respectively (Figure 4A,C). In contrast, pErk was increased by subchronic administration concentration-dependently [F (3,20) = 14.0 (*p* < 0.01)] (Figure 4B).

#### 2.2.2. Effects of QTP on 5-HT7R and Cx43

**Figure 5:**
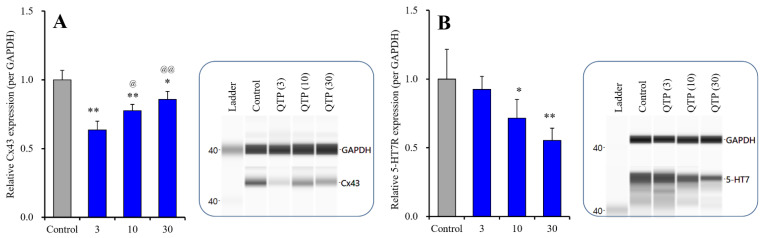
Concentration-dependent effects of subchronic administration (7 days) of QTP on protein expression of Cx43 in the cytosol fraction (**A**) and 5-HT7R in the plasma membrane fraction (**B**) of cortical primary cultured astrocytes. In left side histograms, ordinate: mean ± SD (*n* = 6) of the relative protein level of Cx43 and 5-HT7R per GAPDH. Abscissa: QTP level (μM). * *p* < 0.05, ** *p* < 0.01: relative to control (QTP free), @ *p* < 0.05, @@ *p* < 0.01: relative to 3 μM QTP in panel A by one-way ANOVA with Tukey’s post hoc test. Right side panels indicate their pseudo-gel images, using capillary immunoblotting.

The subchronic administration of QTP decreased 5-HT7R in the plasma membrane fraction, in a concentration-dependent manner [F (3,20) = 9.4 (*p* < 0.01)] (Figure 5B). In contrast to 5-HT7R, expression of Cx43 in the cytosol fraction was also decreased by QTP [F (3,20) = 39.1 (*p* < 0.01)], but the peak reduction in Cx43 level was generated by the lowest level of QTP (3 μM) (Figure 5A).

### 2.3. Effects of QTP on Intracellular cAMP Level in Astrocytes

The concentration-dependent biphasic effects of QTP on pAMPK (upregulation) and Cx43 (downregulation of transcription) appear to be inversely correlated. Transcription of Cx43 is regulated by AMPK signalling via histone deacetylase [38,39,57,58]. AMPK is regulated by cAMP-dependent protein kinase (PKA) and exchange protein directly activated by cAMP (EPAC) [57,59], which is a major second messenger downstream of 5-HT7R [26,60]. Therefore, the concentration-dependent effects of the subchronic administration of QTP on cAMP synthesis were determined.

**Figure 6:**
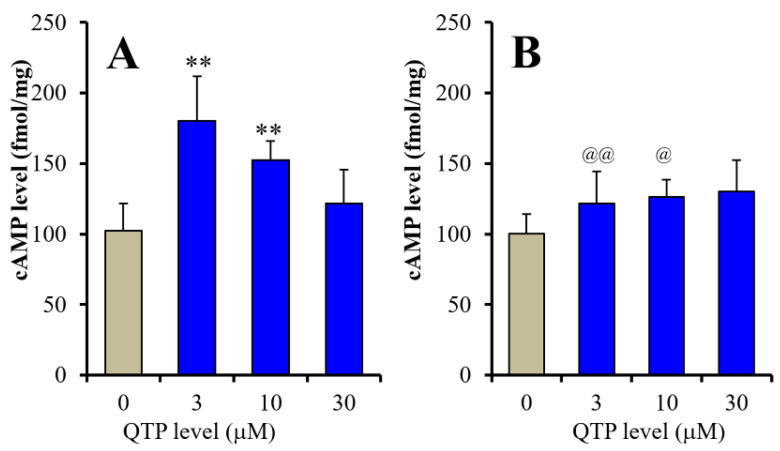
Concentration-dependent effects of subchronic administration of QTP (**A**) and interaction between subchronic administrations of QTP and (2R)-1-[(3-hydroxyphenyl)sulfonyl]-2-[2-(4-methyl-1-piperidinyl)ethyl]pyrrolidine hydrochloride (SB269970: 10 μM) (**B**) on intracellular cAMP level in astrocytes. Ordinates indicate mean ± SD (*n* = 6) of intracellular cAMP level in cultured astrocytes (f mol/mg). ** *p* < 0.01: relative to control (QTP free), @ *p* < 0.05, @@ *p* < 0.01: relative to SB269970 free by two-way ANOVA with Tukey’s post-hoc test.

According to our expectations, QTP biphasically affected cAMP levels in astrocytes [F_QTP_ (3,40) = 12.5 (*p* < 0.01), F_SB269970_ (1,40) = 6.1 (*p* < 0.01), F_QTP*SB269970_ (3,40) = 10.5 (*p* < 0.01)] and its peak concentration was at 3 μM QTP (Figure 6A). A 5-HT7R inverse agonist, (2R)-1-[(3-Hydroxyphenyl)sulfonyl]-2-[2-(4-methyl-1-piperidinyl)ethyl]pyrrolidine hydrochloride (SB269970: 10 μM), supressed the stimulatory effects of 3 μM and 10 μM QTP on cAMP levels without affecting the basal cAMP level (Figure 6B). Therefore, these results suggest that QTP increased cAMP synthesis, but a higher concentration of QTP attenuated its stimulatory effects on cAMP synthesis via 5-HT7R inhibition.

### 2.4. Effects of Subchronically Systemic Administration of QTP in Rat Hypothalamus In Vivo

#### 2.4.1. Effects of Subchronically Systemic Administration of QTP on Expression of pAMPK and Cx43 in the Hypothalamus

AMPK regulates energy homeostasis and the transcription processes of several ion channels via histone deacetylase [38,39,43,57,58]. The resemblance of the effect of QTP on AMPK signalling and the dose-dependent bell-shaped pattern on weight gain suggest the possibility that AMPK signalling contributes to the biphasic weight gain induced by QTP [42]. Therefore, to clarify the possible mechanisms regarding the pathophysiology of QTP on weight gain, the subchronically systemic administration of QTP on hypothalamic AMPK signalling in vivo was evaluated. Previous preclinical studies have reported that the effective dose in the systemic administration of QTP against schizophrenia models was 10 mg/kg/day [17,61,62,63,64]. In particular, 10 mg/kg QTP improved the phencyclidine-induced deficits of prepulse inhibition [61]. Several microdialysis studies also demonstrated that the systemic administration of lower doses of QTP (lower than 5 mg/kg) and a higher dose of QTP (higher than 10 mg/kg) increased norepinephrine and dopamine release via inhibition of monoamine receptors, such as α1A adrenoceptor, 5-HT2AR and D2R [17,62,63,64]. Based on the previous preclinical findings, in the present study, to explore the dose-dependent effects of the systemically subchronic administration of QTP on cyclic adenosine monophosphate (cAMP) levels and protein expression in the rat hypothalamus, rats were subcutaneously administered QTP (10 or 30 mg/kg/day) for 7 days using an osmotic pump (2ML_1, Alzet, Cupertino, CA, USA).

**Figure 7:**
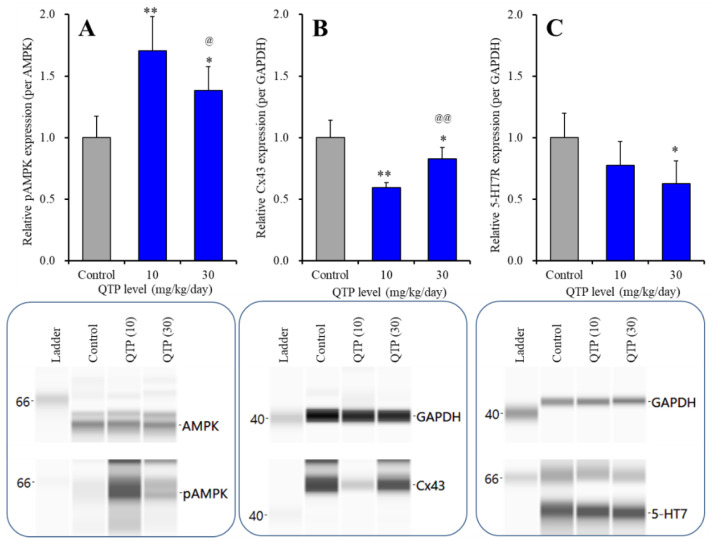
Dose-dependent effects of systemically subchronic administration (7 days) of QTP on protein expression of pAMPK (**A**), Cx43 (**B**) in the cytosol fraction and 5-HT7R (**C**) in the plasma membrane fraction of rat hypothalamus. In upper side histograms, ordinate: mean ± SD (*n* = 6) of the relative protein level of pAMPK per AMPK, Cx43 and 5-HT7R per GAPDH. Abscissa: QTP dose (mg/kg/day). * *p* < 0.05, ** *p* < 0.01: relative to control (QTP free), @ *p* < 0.05, @@ *p* < 0.01: relative to 10 mg/kg/day QTP by one-way ANOVA with Tukey’s post-hoc test. Lower side panels indicate their pseudo-gel images using capillary immunoblotting.

Subchronic administration of QTP (10 and 30 mg/kg/days) for 7 days biphasically affected pAMPK levels in the rat hypothalamus [F (2,15) = 15.6 (*p* < 0.01)] (Figure 7A). The effective dose (10 mg/kg/day) of QTP increased pAMPK, whereas a higher dose (30 mg/kg/day) of QTP also increased pAMPK, but the increase in pAMPK was lower than that induced by 10 mg/kg/day (Figure 7A). The biphasic action of QTP on Cx43 protein expression in the cytosol fraction of the hypothalamus displayed dose-dependent biphasic action [F (2,15) = 24.3 (*p* < 0.01)] (Figure 7B) that was inverse to that on pAMPK. The effective dose (10 mg/kg/day) of QTP decreased Cx43 expression, whereas a higher dose (30 mg/kg/day) of QTP also decreased Cx43 expression but the level of Cx43 was higher than that at 10 mg/kg/day (Figure 7B). In contrast to pAMPK and Cx43, subchronic administration of QTP decreased 5-HT7R in the plasma membrane of the hypothalamus [F (3,20) = 5.8 (*p* < 0.05)] (Figure 7C). Subchronic administration of 30 mg/kg/day QTP decreased 5-HT7R, but 10 mg/kg/day QTP did not affect it (Figure 7C).

#### 2.4.2. Effects of Subchronically Systemic Administration of QTP on cAMP Level in the Hypothalamus

**Figure 8:**
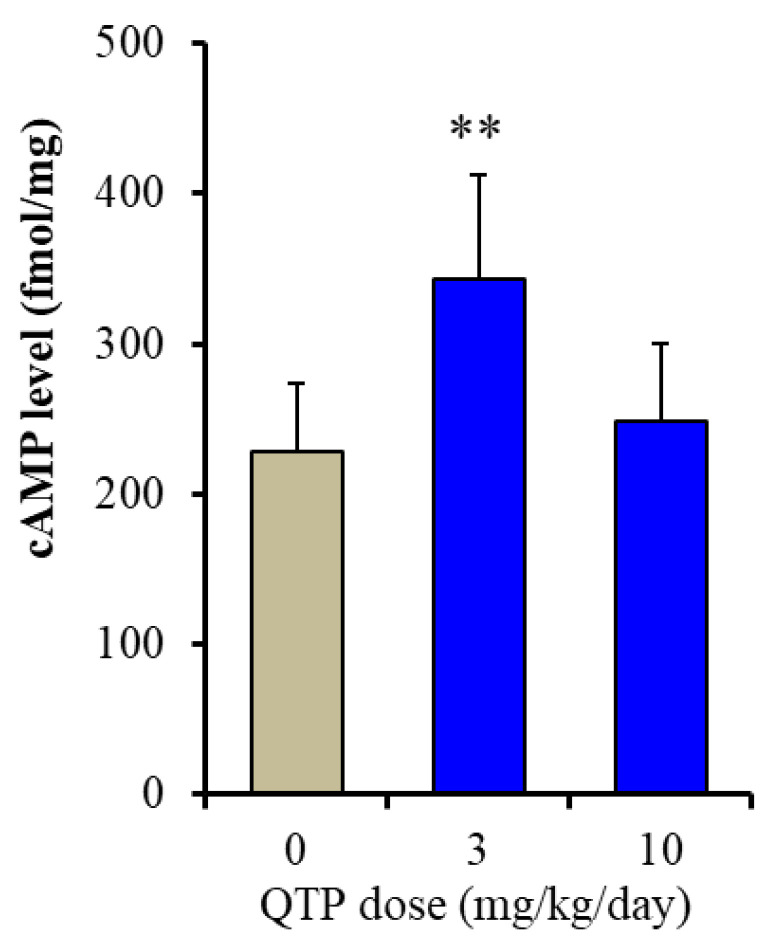
Effects of subchronically systemic administration of QTP on cAMP levels in the rat hypothalamus. Ordinates indicate mean ± SD (n  =  6) of cAMP level in rat hypothalamus (fmol/mg). Abscissa: QTP dose (mg/kg/day). ** *p* < 0.01: relative to control (QTP free) by one-way ANOVA with Tukey’s post-hoc test.

To clarify the mechanisms of the discrepant effects between 10 and 30 mg/kg/day of QTP on AMPK signalling, we explored the effect of the subchronic administration of QTP for 7 days on cAMP synthesis in the rat hypothalamus. Subchronic administration of the effective dose of QTP (10 mg/kg/day) for 7 days increased cAMP levels in the rat hypothalamus, whereas that of 30 mg/kg/day QTP did not affect them (Figure 8).

## 3. Discussion

### 3.1. Effects of Subchronic Administration of QTP on 5-HT7R

QTP is considered to be a low-affinity 5-HT7R antagonist, but the detailed function of QTP on 5-HT7R remains to be clarified [15]. It has been already demonstrated that several mood-stabilising atypical antipsychotics and antidepressants, such as clozapine, olanzapine, lurasidone, brexpiprazole and vortioxetine, are 5-HT7R inverse agonists, since these agents acutely inhibited 5-HT7R and subchronically downregulated/desensitised 5-HT7R, similar to selective 5-HT7R inverse agonist, SB269970 [31,38,39,44]. Therefore, the present study indicated the possibility that QTP is not a 5-HT7R antagonist but is a candidate 5-HT7R inverse agonist, since QTP acutely inhibited 5-HT7R function and subchronically downregulated 5-HT7R. The activation of 5-HT7R enhances adenylyl cyclase via activation of the Gsα protein [26]. Indeed, both acute and subchronic administration of SB269970 inhibits adenylyl cyclase [31,37,38,39]. In the present study, subchronic application of a therapeutically relevant concentration of QTP (3 μM) increased cAMP levels without affecting 5-HT7R expression in the plasma membrane fraction. In contrast to the therapeutically relevant concentration, subchronic application of a higher concentration of QTP (10 and 30 μM) concentration-dependently downregulated 5-HT7R, whereas 10 μM and 30 μM QTP increased and did not affect the cAMP level, respectively. These results suggest that the therapeutically relevant concentration of QTP could not affect 5-HT7R due to its low affinity to 5-HT7R, but a higher concentration of QTP concentration-dependently suppressed 5-HT7R function. In the present study, a potent 5-HT7R inverse agonist, SB269970, attenuated the increasing cAMP level induced by 3 μM and 10 μM QTP but did not affect that induced by 30 μM QTP. These results suggest that the 5-HT7R inverse agonistic action of QTP was not able to act at a therapeutically relevant concentration, but a higher concentration of QTP (30 μM) suppresses 5-HT7R.

### 3.2. Effects of Subchronic Administration of QTP on Intracellular Signalling in Astrocytes

The present study also demonstrated the concentration-dependent biphasic effects of QTP on intracellular signalling, including Akt, Erk and AMPK, but each type of signalling exhibited a specific QTP concentration dependency. Indeed, the peak concentrations of QTP for AMPK, Akt and Erk were lower than 3 μM and 10 μM and higher than 30 μM, respectively. It is well known that Erk and Akt play important roles in the pathophysiology of mood disorders and cognitive impairments [65], since D2R, 5-HT1A and 5-HT7R regulate both Akt and Erk signalling’s [31,66,67,68,69,70].

It has been considered that Akt signalling is one of the key players in the pathophysiology of schizophrenia, since Akt-deficit mice showed impaired prepulse inhibition [71]. Long-term administration of antipsychotics, such as haloperidol, clozapine, olanzapine, QTP, risperidone and zotepine, enhance Akt signalling [18,40,69,70,71] due to Akt dephosphorylation [69]. Selective 5-HT reuptake inhibitors also enhance Akt signalling via activation of 5-HT1AR [72]. Similar to Akt signalling, several antipsychotics, such as haloperidol, clozapine and risperidone, activate Erk signalling [67,68], whereas these effects were not observed in D2R-deficit mice [66]. Additionally, activation of 5-HT1AR and inhibition of 5-HT2AR enhance Erk signalling [73,74]. Considering these previous findings, the antagonistic actions toward D2R and 5-HT2AR of QTP activate both Akt and Erk signalling.

In contrast to Akt and Erk signalling, the effects of QTP on AMPK signalling need detailed discussion. It has been established that AMPK plays fundamental roles in the regulation of energy homeostasis/metabolism, since AMPK signalling is activated by increasing energy expenditure, such as decreasing glucose and ATP, resulting in the activation of hepatic gluconeogenesis and glycogenolysis [75]. It is well known that several antipsychotics enhance AMPK signalling due to H1R inhibition [43,76]. The high-affinity H1R antagonists clozapine and olanzapine are some of the strongest AMPK activators, whereas the stimulatory effects of clozapine on AMPK signalling were not observed in H1R-deficit mice [76]. Furthermore, the histaminergic stimulant betahistine suppressed the olanzapine-induced activation of AMPK signalling [75]. Based on these findings, the activation of AMPK signalling induced by H1R antagonistic antipsychotics has been considered to be important in the pathophysiology of antipsychotic-induced weight gain and the development of metabolic complications [43]. According to this hypothesis, it was reasonable that the therapeutically relevant concentration of QTP enhanced AMPK signalling due to the high-affinity H1R antagonistic action of QTP (Ki = 11 nM). With regard to the biphasically concentration-dependent stimulatory effects of QTP on AMPK signalling (bell-shaped pattern), the therapeutically relevant concentration (3 μM) enhanced AMPK signalling but the stimulatory effects of the higher concentrations (10 and 30 μM) were attenuated compared to the therapeutically relevant concentration, which is consistent with the dose dependency of QTP on weight gain [14]. Exploring the mechanism by which the activation of AMPK at higher concentrations of QTP diminishes compared to that of the therapeutic concentration of QTP is clinically important for appropriate dose determination.

Both lurasidone and brexpiprazole have been evaluated as the safest options in patients with the risk of developing metabolic complications and weight gain, since these antipsychotics are listed among the best atypical antipsychotics associated with metabolic outcomes [14,42]. Lurasidone has the lowest affinity to H1R antipsychotics (Ki = >1000 nM); however, brexpiprazole is a H1R antagonist (Ki = 19 nM), but is a low-risk antipsychotic for metabolic complications [14,42]. Therefore, the contradiction between lurasidone and brexpiprazole regarding the low risk for metabolic complications cannot be fully explained by their affinity to H1R. Recently, the possible 5-HT7R inverse agonistic actions of lurasidone and brexpiprazole, which acutely inhibit but subchronically downregulate 5-HT7R, contributed to the persistent prevention of the activation of AMPK signalling via suppression of adenylyl cyclase activity [38,39]. Therefore, a therapeutically relevant concentration of QTP enhances AMPK signalling induced by H1R inhibition, whereas at a higher concentration of QTP, the 5-HT7R inverse agonistic action probably is actuated, resulting in the attenuation of its stimulatory effects on AMPK signalling induced by H1R inhibition.

### 3.3. Dose-Dependent Clinical Action of QTP

The major purpose of this study was to explore the pathophysiology of a high dose of QTP (higher than 600 mg/day), which is mainly related to the antipsychotic and anti-manic effects and reduction in the risk of weight gain and metabolic complications compared to a lower dose of QTP (300~600 mg/day). For this purpose, therefore, the present study also determined the dose-dependent effects of QTP on AMPK signalling in the hypothalamus, since the enhancement of AMPK signalling in the hypothalamus is one of the major mechanisms of weight gain induced by several antipsychotics [38,39,43]. According to our expectations, the therapeutically relevant dose of QTP activated hypothalamic AMPK, but a high dose of QTP attenuated the enhanced hypothalamic AMPK signalling compared to the therapeutically relevant dose. This attenuation is possibly associated with similar mechanisms, namely the downregulation of 5-HT7R and attenuation of cAMP synthesis observed in astrocytes. Therefore, the results from the in vitro experiment using cultured astrocytes can be used to interpret the mechanism involved in clinical findings that the 5-HT7R inverse agonistic action of QTP plays important roles in the biphasically dose-dependent effects of QTP (bell-shaped pattern) on weight gain [14]. In other words, the 5-HT7R inverse agonistic action probably contributes to the prevention of antipsychotic-induced weight gain [38,39,44].

The 5-HT7R inverse agonistic action of QTP is also interesting in terms of the pathophysiology of mood disorders associated with tripartite synaptic transmission. It has been reported that the enhancement of astroglial Cx43-containing hemichannels plays important roles in mood-stabilising action, since several mood-stabilising atypical antipsychotics, such as clozapine, quetiapine, zotepine and brexpiprazole, enhance astroglial L-glutamate release via activated Cx43-containing hemichannels [18,38,39,40]. The therapeutically relevant concentration of QTP enhanced astroglial L-glutamate release via the astroglial Cx43-containing hemichannel [18]. In the present study, QTP increased astroglial D-serine release through the activated hemichannel. D-Serine is a gliotransmitter and an endogenous, potent co-agonist with the NMDA glutamate receptor. It is known that the NMDA receptor antagonist esketamine has been approved for the treatment of conventional monoaminergic antidepressant-resistant depression [77]; however, conversely, both clinical and preclinical investigations revealed that D-serine exhibited antidepressant action [78]. Contrarily, an NMDA receptor agonist generates severe cognitive impairments, whereas D-serine is considered to be a candidate cognitive enhancer [79]. The mechanisms of the similar antidepressive efficacies and opposite effects against cognition among NMDA receptor agonists and antagonists remain to be clarified [80], whereas the stimulatory effects of QTP on astroglial D-serine release through astroglial hemichannels are possibly involved in the clinical efficacy, antidepressive and cognitive enhancement effects of QTP. Trafficking of Cx43 to the plasma membrane is positively regulated by Akt signalling [27,28,31,37,52], which is activated by a therapeutically relevant concentration of QTP. However, therapeutically relevant concentration of QTP unexpectedly did not increase Cx43 protein expression in the astroglial plasma membrane, whereas, under histone deacetylate inhibition, QTP increased Cx43 expression in the plasma membrane [18]. The present study probably revealed the underlying mechanism of the contradiction of therapeutically relevant concentration of QTP on Cx43 protein expression in the cytosol and plasma membrane. In the present study, the therapeutically relevant concentration of QTP enhanced and decreased AMPK signalling and Cx43 protein expression in the astroglial cytosol fraction. The transcription of Cx43 is suppressed by histone deacetylase, which is activated by AMPK signalling [57]. In other words, at the therapeutically relevant concentration of QTP, it appeared that there was no change in Cx43 expression in the plasma membrane, due to the combination of the contrasting effects between the suppression of Cx43 protein synthesis and enhancement of trafficking Cx43 to the plasma membrane. QTP has been evaluated as a wide-spectrum mood-stabilising antipsychotic, and QTP monotherapy is effective in the treatment of acute mania and prevention of recurrence of mania and bipolar depression [7,9,10]. Additionally, the efficacy of the combination therapy with valproate and QTP has also been established regarding its effectiveness for the treatment of various mood disorders [11,81]. Taken together with this clinical evidence, the effectiveness of valproate as an adjunct therapy to QTP is possibly modulated by the relative enhancement of Cx43 function due to the activation of Cx43 transcription induced by histone deacetylase inhibition.

The high seizure risk during QTP treatment is well known [82,83], whereas, contrary to clozapine [84], the dose dependence of the seizure risk of QTP remains to be clarified. For QTP-induced seizure, the timing appears to range from several hours to one day after the supratherapeutic dose of QTP is administered [85,86,87]. Tmax and T1/2 values of QTP are approximately 1.5 and 6 h, respectively [88]. Therefore, the delay onset of QTP-induced seizure following QTP intake is peculiar in light of the pharmacokinetics of QTP. In other words, the pathophysiology of QTP-induced seizure is probably not attributed to the receptor binding profiles of QTP. Considering the turnover of Cx43 (several hours), at least more than several hours after QTP administration are required to increase the release of excitatory gliotransmitters, including L-glutamate and D-serine, through activated hemichannels via the activation of various processes, such as transcription, folding, trafficking and activation of Cx43. Recent clinical physiology explored the impacts of high-frequency oscillations on brain function and identified that high-frequency oscillations exhibit both physiological and pathological functions in a frequency-dependent manner. Physiological high-frequency oscillation, ripple-burst (80~250Hz), which is synchronised with the sleep-spindle burst, plays important roles in cognitive function, whereas pathological fast ripple-burst (250~500 Hz) contributes to epileptic discharge and neuronal damage [51,89]. The results that QTP could not directly activate the astroglial hemichannel, but enhanced astroglial excitatory gliotransmitters (L-glutamate and D-serine) release through the activated hemichannel, suggest the possible pathophysiology of a delayed-onset QTP-induced seizure. A recent study reported that ripple burst could be involved in ictogenesis in the networks that have acquired vulnerability to epileptogenesis [49,51]. Considering these previous findings, this study can propose a working hypothesis regarding delayed-onset QTP-induced seizure. A therapeutic dose of QTP suppresses the excessive functioning of the Cx43-containing hemichannel via inhibition of Cx43 transcription by the activation of AMPK signalling, whereas a supratherapeutic dose of QTP generates an excessive increase in the Cx43-containing hemichannel in the plasma membrane via the combination of inhibited AMPK and activated Erk signalling. The increased Cx43-containing hemichannel in the plasma membrane persists for at least several hours (half-life of Cx43 in the plasma membrane). During this condition, the supratherapeutic concentration of QTP is rapidly decreased due to the short T1/2 kinetics feature of QTP, resulting in the generation of disinhibition. Therefore, the positive interaction between disinhibition and increasing Cx43-containing astroglial hemichannels plays important roles in the pathophysiology of delayed-onset QTP-induced seizures [85].

## 4. Materials and Methods

### 4.1. Chemical Agents and Drug Administration

Quetiapine fumarate (QTP), selective Cx43 inhibitor, TAT-conjugated Gap19 (Gap19), Akt inhibitor, 10-[4-(N,N-diethylamino)butyl]-2-chlorophenoxazine hydrochloride (DEBC) and AMPK inhibitor, dorsomorphin hydrochloride were obtained from Funakoshi (Tokyo, Japan). Erk inhibitor, 5-(2-Phenyl-pyrazolo [1,5-a]pyridin-3-yl)-1H-pyrazolo[3,4-c]pyridazin-3-ylamine (FR180204) was obtained from Tokyo Chemical Industry (Tokyo, Japan). 5-HT7R inverse agonist, (2R)-1-[(3-Hydroxyphenyl)sulfonyl]-2-[2-(4-methyl-1-piperidinyl)ethyl] pyrrolidine hydrochloride (SB269970), were obtained from Cosmo-Bio (Tokyo, Japan). All agents were prepared on the day of the experiment. Gap19, DEBC, SB269970 and dorsomorphin were directly dissolved in Dulbecco’s modified Eagle’s medium containing 10% foetal calf serum (fDMEM) or artificial cerebrospinal fluid (ACSF). QTP and FR180204 were initially dissolved in dimethyl sulfoxide at 25 mM. The final dimethyl sulfoxide concentration was lower than 0.1% (*v*/*v*).

The therapeutic-relevant serum concentration of QTP was reported to be approximately ranged from 0.3 μM to 3 μM. Based on the clinical findings, in the present study, cultured astrocytes were administrated by 3, 10 and 30 μM QTP for 7 days. Previous studies have reported that the effective dose of systemic administration of QTP was 10 mg/kg/day. Based on the previous reports, in the present study, to explore the dose-dependent effects of systemically subchronic administration of QTP on cyclic adenosine monophosphate (cAMP) levels and protein expression in the rat hypothalamus, rats were subcutaneously administered by QTP (10 or 30 mg/kg/day) for 7 days using an osmotic pump (2ML_1, Alzet, Cupertino, CA, USA).

### 4.2. Preparation of Primary Astrocyte Culture

Experimental procedures and animal care in this study were performed according to the ethical guidelines established by the Institutional Animal Care and Use Committee at Mie University, Japan (No. 2019-3) and the ARRIVE (Animal Research: Reporting of In vivo Experiments) guidelines. The protocol of astrocytes preparation was mainly followed the previous study. Each pregnant Sprague–Dawley rat (SLC, Shizuoka, Japan) was housed individually in a cage (in air-conditioned rooms: temperature, 22 ± 2 °C) with 12 h light/dark cycle. Each rat freely accesses to food and water.

Neonatal Sprague–Dawley rats (n = 42), which were sacrificed by decapitation at 0–48 h of age. The cerebral hemispheres were removed under the dissecting microscope. The cerebral tissue was chopped by fine pieces using scissors, and then triturated briefly with a micropipette. The suspension was filtered using 70 µm nylon mesh (BD, Franklin Lakes, NJ, USA) and then centrifuged at 200 rpm. The pellets were resuspended in 10 mL Dulbecco’s modified Eagle’s medium (D6546; Sigma-Aldrich, St. Louis, MO, USA) containing 10% foetal calf serum (fDMEM). The day after culturing for 14 days (DIV14), contaminated non-astrocytes were removed by shaking in a standard incubator for 16 h at 200 rpm. Astrocytes were removed from flasks by trypsinisation and seeded directly onto a translucent polyethylene terephthalate (PET) membrane (1.0 μm) with 24 well plates (BD) at a density of 100 cells cm^2^. The culture medium (fDMEM) was changed twice a week, and QTP and other target agents were administered subchronically (for 7 days, DIV21–28).

### 4.3. Artificial Ripple-Burst Evoked Stimulation

To clarify the physiological electrostimulation on astroglial transmitter release, in the present study, cultured astrocytes were electrically stimulated using ripple-burst evoked stimulation, using a busdrive amplifier (SEG-3104MG, Miyuki Giken, Tokyo, Japan). Sleep spindle bursts are generally considered to be coupled with ripple-bursts, as determined using wide-band electrocorticogram. Recently, ripple-bust plays important roles in the he sleep-dependent memory consolidation. Ripple-burst evoked stimulation was set at a square-wave direct current pulse output, with a magnitude of 300 mV/mm^2^. A set of ripple-burst evoked stimulations was composed of 10 stimuli at 200 Hz and 10 bursts (50% duty cycle) at burst intervals of 100 ms per 1 s. These stimulation patterns of ripple-burst evoked stimulations were regulated using LabChart version 8.2 software (AD Instruments, Dunedin, New Zealand). Cultured astrocytes were stimulated by ripple-burst evoked stimulation in artificial cerebrospinal fluid (ACSF: comprised NaCl 150.0 mM, KCl 3.0 mM, CaCl_2_ 1.4 mM, MgCl_2_ 0.8 mM and glucose 5.5 mM, and was buffered to pH 7.3 with 20 mM HEPES buffer).

### 4.4. Extraction of Preteins and cAMP from Cultured Astrocytes and Rat Hypothalamus

On DIV28, before extraction, the cultured astrocytes were washed out by ACSF. After the systemically subchronic administration of effective doses of QTP, the rat hypothalamus was dissected according to the method of Glowinski and Iversen. To analyse the protein levels using capillary immunoblotting system, after the washout, the cytosol and plasma membrane fractions of cultured astrocytes and rat dissected hypothalamus were extracted by Minute Plasma Membrane Protein Isolation Kit (Invent Biotechnologies, Plymouth, MN, USA). To analyse the intracellular cAMP level, after the washout, the cultured astrocytes and rat dissected hypothalamus were placed into respective 0.5 mL and 1.5 mL microtubes, and homogenised by ultrasonic cell disrupter (VP-050N, Taitec, Koshigaya, Japan) in chilled 0.1 N HCl, and then the homogenised sample was centrifuged at 10,000× *g* for 20 min at 4 °C. Filtered aliquots (5 μL) were injected into the ultra-high-performance liquid-chromatography (UHPLC) with mass spectrometry system (LC-MS).

### 4.5. Capillary Immunoblotting Analysis

The protein expression level was determined using capillary immunoblotting Wes system (ProteinSimple, Santa Clara, CA, USA), according to mainly instruction protocol. The lysates of the primary cultured astrocytes and hypothalamus were mixed with a master mix solution (ProteinSimple). The mixture (final concentration of 1× sample buffer, 1× fluorescent molecular weight marker and 40 mM dithiothreitol) was heated at 95 °C for 5 min. The samples, blocking reagents, primary antibodies, HRP-conjugated secondary antibodies, chemiluminescent substrate (SuperSignal West Femto; Thermo Fisher Scientific, Waltham, MA, USA) and separation/stacking matrices were also dispensed into the designated 25 well plate. After loading, separation electrophoresis and immunodetection steps were performed in the capillary system, which was fully automated at room temperature, and the instrument’s default settings were used. Capillaries were first filled with a separation matrix, followed by a stacking matrix, with approximately 40 nL of the sample used for loading. During electrophoresis process, the proteins were separated by molecular weight through the stacking/separation matrices at 250 V for 40–50 min and then immobilised on the capillary wall using proprietary photo-activated capture chemistry. The capillaries were incubated with blocking reagent for 15 min, and the target proteins were probed with primary antibodies, followed by HRP-conjugated secondary antibodies (Anti-Rabbit IgG HRP, A00098, 10 μg/mL, GenScript, Piscataway, NJ, USA). Antibodies against GAPDH (NB300-327, 1:300, Novus Biologicals, Littleton, CO, USA), connexin43 (C6219, 1:100, Sigma-Aldrich), 5-HT1AR (NBP2-21590, 1:00, Novus Biologicals), 5-HT7R (NB100-56352, 1:00, Novus Biologicals), Erk (AF1576, 10 μg/mL, R&D systems, Minneapolis, MN, USA), phosphorylated-Erk (AF1018, 5 μg/mL, R&D Systems), Akt (AF1775, 1 μg/mL, R&D Systems), phosphorylated-Akt (AF877, 5 μg/mL, R&D Systems), AMPKα (2603, 1:50, Cell Signalling Technology, Danvers, MA, USA) and phosphorylated-AMPKα (2535, 1:50, Cell Signalling Technology) were diluted in an antibody diluent (Immuno Shot Platinum, CosmoBio, Tokyo, Japan).

### 4.6. UHPLC and LC-MS

Extracellular levels of D-serine were analysed by UHPLC (xLC3185PU, Jasco, Tokyo, Japan) equipped with fluorescence detector (xLC3120FP, Jasco): excitation/emission wavelengths were set at 280/455 nm. The sample was derivatised with isobutyryl-L-cysteine and o-phthalaldehyde. The derivatised samples (5 μL aliquots) were automatically injected by autosampler (xLC3059AS, Jasco), and separated by analytical column (YMC Triat C18, particle 1.8 μm, 50 × 2.1 mm, YMC, Kyoto, Japan) maintained at 500 μL/min and 45 °C. A linear gradient elution program was used for over 10 min with mobile phases: A (0.05 M citrate buffer, pH 5.0) and B (0.05 M citrate buffer containing 30% acetonitrile and methanol, pH 3.5).

The cAMP levels in rat hypothalamus were analysed by UHPLC (Acquity UPLC H-Class system; Waters, Milford, MA, USA) with mass spectrometry (Acquity SQ detector; Waters). The samples (5 μL aliquots) were automatically injected by autosampler (Acquity UPLC Sample Manager FTN; Waters), and separated by analytical column, graphite carbon column (particle 3 μm, 150 × 2.1 mm; Hypercarb, Thermo) maintained at 450 μL/min at 40 °C. A linear gradient elution programme was used for over 10 min with mobile phases: A (1 mM ammonium acetate buffer, pH 11) and B (100% acetonitrile). The nitrogen flows of the desolvation and cone were set at 750 and 5 L/h, respectively. The desolvation temperature was set at 450 °C. The cone voltage for the determination of cAMP (m/z = 330.3) was 42 V.

### 4.7. Data Analysis

According to previous studies, all experimental designs in this study were set at equally sized animal groups (*n* = 6), without conducting formal power analyses. All data are represented as mean ± standard deviation (SD), and *p* < 0.05 (two-tailed) was considered statistically significant. Agent concentrations of subchronic administration were adopted according to previous studies. To randomise and blind the determination of levels of D-serine, cAMP and protein expression, the samples were set on autosampler according to the random number tables.

All statistical analyses in this study were performed using Bell Curve for Excel ver3.2 (Social Survey Research Information Co., Ltd., Tokyo, Japan). The concentration-dependent and dose-dependent effects of QTP on cAMP levels and protein expression levels were analysed using one-way analysis of variance (ANOVA) with Tukey’s multiple comparison. The interaction between QTP and SB269970 on cAMP level in the astrocytes was analysed by two-way ANOVA with Tukey’s multiple comparison. Effects of inhibitors of Cx43, Akt, Erk and AMPK on ripple-burst evoked astroglial D-serine release were analysed by student T-test. Concentration-dependent effects of subchronic administrations of QTP on astroglial D-serine release were analysed by one-way ANOVA with Tukey’s multiple comparison. Interaction between QTP and inhibitors of Akt, Erk and AMPK on astroglial D-serine release were analysed by two-way ANOVA with Tukey’s multiple comparison. The data and statistical analysis comply with the recommendations of the British Journal of Pharmacology on experimental design and analysis in pharmacology.

### 4.8. Nomenclature of Targets and Ligands

Key protein targets and ligands in this report are hyperlinked to corresponding entries in http://www.guidetopharmacology.org accessed on 19 July 2022, the common portal for data from the IUPHAR/BPS Guide to PHARMACOLOGY, and are permanently archived in the Concise Guide to PHARMACOLOGY 2021/22.

## 5. Conclusions

The present study determined the effects of the subchronic administration of a therapeutically relevant and supratherapeutic concentration/dose of QTP on astroglial signalling associated with 5-HT7R, to explore the mechanisms underlying mood-stabilising antipsychotic effects and metabolic complications of QTP. Subchronic administrations of QTP downregulated 5-HT7R concentration-dependently, whereas the therapeutically relevant concentration of QTP (3 μM) did not affect 5-HT7R expression. The fact that QTP acutely inhibited 5-HT7R function but downregulated 5-HT7R suggests the possibility that QTP is a candidate low-affinity 5-HT7R inverse agonist, similar to clozapine and olanzapine. QTP enhanced astroglial Erk signalling concentration-dependently, but 3 μM QTP did not affect Erk signalling. QTP also enhanced astroglial D-serine release, cAMP synthesis and Akt and AMPK signalling, displaying a bell-shaped pattern. These complicated concentration-dependent effects of the subchronic administration of QTP were observed in the rat hypothalamus in vivo. Therefore, these results suggest that the therapeutically relevant concentration/dose of QTP cannot affect 5-HT7R, but a higher concentration/dose of QTP suppresses 5-HT7R function via the acute inhibition and subchronic downregulation of 5-HT7R. Importantly, the adverse reactions to QTP, namely weight gain and metabolic complications, showed bell-shaped responses to the QTP dose, peaking at 600 mg/day. Therefore, the 5-HT7R inverse agonistic action probably plays important roles in the prevention of some adverse reactions to QTP, such as weight gain and metabolic complications.

## Figures and Tables

**Table 1:** Receptor binding profiles of quetiapine and other mood stabilizing atypical antipsychotics.

Transmitter	Receptor	QTP	NQTP	Brex	CLZ	LUR	ZTP
Histamine	H1R	11	3.5	19	1.13	>1000	3.21
Serotonin	5-HT1AR	432	45	0.12	124	6.8	471
(5-HT)	5-HT2AR	100	48	0.47	5.4	2.0	2.7
	5-HT7R	307	76	3.7	18.0	0.5	12.0
Norepinephrine	α1A	22	144	3.8	1.62	47.9	7
	α2A	>1000	237	15	37	40.7	180
Dopamine	D1R	712	214	160	266	262	71.0
	D2R	245	196	0.3	157	1.7	25.0
	Reference	[15]	[15]	[21]	[22,23]	[24]	[25]

Quetiapine (QTP), QTP metabolite, norquetiapine (NQTP), brexpiprazole (Brex), clozapine (CLZ), lurasidone (LUR), zotepine (ZTP) against serotonin (5-HT) type 1A (5-HT1AR), type 2A (5-HT2AR), type 7 (5-HT7R), dopamine receptors type 1 (D1R) and 2 (D2R) and adrenoceptor type α1A (α1A) and α2A (α2A) and histamine type 1 (H1R) receptor. Data are equilibrium constant (Ki) values (nM).

## Data Availability

The data that support the findings of this study are available from the corresponding author upon reasonable request. Some data may not be made available because of ethical restrictions.
